# Data on the thermal properties of soil and its moisture content

**DOI:** 10.1016/j.dib.2018.02.018

**Published:** 2018-02-13

**Authors:** K.D. Oyeyemi, O.A. Sanuade, M.A. Oladunjoye, A.P. Aizebeokhai, A.A. Olaojo, J.O. Fatoba, O.M. Olofinnade, W.A. Ayara, O. Oladapo

**Affiliations:** a Department of Physics, Covenant University, Ota, Ogun State, Nigeria; b Department of Geosciences, King Fahd University of Petroleum & Minerals, Dhahran, Saudi Arabia; c Department of Geology, University of Ibadan, Nigeria; d Department of Earth Sciences, Ajayi Crowther University, Oyo, Nigeria; e Department of Geophysics, Federal University Oye-Ekiti, Ekiti State, Nigeria; f Department of Civil Engineering, Covenant University, Ota, Ogun State, Nigeria

## Abstract

The dataset contains thermal properties of soil such as thermal conductivity, thermal diffusivity, temperature and specific heat capacity in an agricultural farm within the University of Ibadan, Ibadan, Nigeria. The data were acquired in forty (40) sampling points using thermal analyzer called KD-2 Pro. Soil samples taken at these sampling points were analyzed in the laboratory for their moisture content following the standard reference of American Association of State Highway and Transport Officials (AASHTO) T265. The data were acquired within the first and second weeks in the month of April, 2012. Statistical analyses were performed on the data set to understand the data. The data is made available publicly because thermal properties of soils have significant role in understanding the water retention capacity of soil and could be helpful for proper irrigation water management.

**Specifications Table**

**Table:** 

Subject area	*Earth, Environment and Planetary science*
More specific subject area	*Thermal Physics*
Type of data	*Tables and figures*
How data was acquired	*KD-2 Pro thermal Analyzer using SH-1 thermal sensor was used to determine the thermal properties at each sampling point, moisture contents of soil samples were equally determined in the laboratory.*
Data format	*Raw and Analyzed*
Experimental factors	*The top of the ground was scooped before measuring thermal properties to mitigate the effect of top layer. The thermal sensor was calibrated using a two-hole Delrin block, the thermal sensor was then correctly placed into the soil and the dual needle was maintained parallel to each other during insertion into the ground.*
Experimental features	*Thermal properties including thermal conductivity and diffusivity, and specific heat of soil were measured. Moisture contents were also measured in the laboratory*
Data source location	*Agricultural farm in University of Ibadan, Ibadan, Nigeria. The study area for the data acquisition is within latitude 7°26′.8020′′ – 7°26′.9320′′ and longitude 3°53′.7230′′ – 3°54′.0000′′*
Data accessibility	*The Data are available within this article*

**Value of the data**

• The dataset can be used to monitor soil moisture content.
• The knowledge of the dataset can help to improve irrigation scheduling in the area.
• The knowledge of the irrigation scheduling would help to optimize water usage for improved crop productivity.
• The dataset would help farmers to save cost.
• The dataset could also be used for academic purposes to understand the applications of thermal properties of soil(s). Several similar Researches to this data article can be found in [1], [2], [3], [4], [5], [6], [7], [8], [9], [10], [11], [12], [13].

## 1 Data

The dataset contains thermal properties of soil and their moisture contain in an agricultural farm within University of Ibadan, Ibadan, Nigeria. These thermal properties include thermal conductivity, thermal diffusivity, temperature and specific heat capacity. The data also contain moisture contents that were measured in the laboratory following the standard reference of American Association of State Highway and Transport Officials (AASHTO) T265 [14] and are shown in Table 1. The understanding of these properties would help in proper irrigation planning for water management which in turn would help to optimize water usage in improving crop productivity. The statistical analyses to further understand the statistical distribution of the data are shown in Table 2.

**Table 1:** Thermal properties and moisture content of soil.

Longitude (X)	Latitude (Y)	Elevation (Z)	Thermal Conductivity (W/mK)	Thermal Diffusivity (mm^2^/s)	Specific heat (mJ/m^3^ K)	Temperature (°C)	Moisture content (m^3^/m^3^
3.893417	7.453417	162	1.379	0.651	2.241	27.500	0.174
3.893917	7.453889	186	1.630	0.874	1.866	27.550	0.187
3.893917	7.453667	191	1.220	0.605	2.018	28.540	0.171
3.893944	7.453389	192	1.899	0.746	2.546	29.170	0.192
3.893528	7.453444	186	1.695	0.770	2.201	28.730	0.188
3.893306	7.45375	165	1.081	0.680	1.615	28.640	0.169
3.893694	7.453417	206	1.769	0.885	2.003	31.350	0.191
3.894528	7.453472	190	1.999	0.797	2.511	29.330	0.194
3.894472	7.45375	203	1.122	0.683	1.643	30.770	0.170
3.8945	7.453833	183	2.057	0.926	2.221	30.060	0.198
3.893389	7.453861	190	2.151	0.914	2.352	25.820	0.223
3.893389	7.453694	202	1.358	0.596	2.279	26.490	0.211
3.893417	7.453444	206	1.225	0.636	1.926	26.030	0.226
3.89325	7.453444	199	1.809	0.822	2.201	27.950	0.218
3.893111	7.453444	194	1.979	0.702	2.821	30.340	0.209
3.893083	7.453694	196	1.335	0.761	1.754	29.250	0.216
3.893222	7.453917	193	1.372	1.000	1.372	28.690	0.199
3.893361	7.454139	179	1.285	0.757	1.696	28.970	0.194
3.893333	7.454222	159	1.707	0.723	2.362	29.540	0.210
3.893361	7.453556	207	1.789	0.884	2.024	29.550	0.213
3.892167	7.453889	188	1.691	0.980	1.725	29.650	0.162
3.892056	7.545111	200	1.184	0.655	1.808	29.740	0.159
2.891444	7.454	199	1.450	0.820	1.768	29.160	0.155
3.8915	7.453861	194	1.511	0.797	1.897	27.490	0.155
3.89175	7.454	192	1.424	0.816	1.746	30.140	0.151
3.891917	7.453972	190	1.810	0.802	2.256	28.970	0.152
3.891639	7.453944	190	1.499	0.698	2.147	30.330	0.146
3.898389	7.44675	191	1.096	0.354	1.431	23.820	0.152
3.898111	7.446944	197	1.216	0.612	1.988	24.260	0.163
3.89775	7.447111	198	1.254	0.513	1.860	23.140	0.168
3.897222	7.447528	199	1.091	0.538	2.028	24.690	0.203
3.896833	7.44775	200	1.018	0.486	2.093	25.760	0.210
3.897	7.447972	196	0.493	0.339	1.453	29.170	0.212
3.897417	7.447861	202	0.800	0.502	1.595	32.300	0.198
3.89775	7.447611	203	1.429	0.487	2.936	24.450	0.209
3.898194	7.447278	204	1.773	0.645	2.748	25.010	0.201
3.898583	7.447056	191	1.167	0.353	3.308	24.910	0.198
3.898722	7.447472	195	0.483	0.381	1.266	26.270	0.203
3.898417	7.447694	199	0.588	0.315	1.866	24.010	0.206
3.898	7.447889	197	1.594	0.351	4.547	23.210	0.199

**Table 2:** Summary of the descriptive statistical analyses of the data.

**Variable**	**Mean**	**StDev**	**Variance**	**CoefVar**	**Minimum**	**Q1**	**Median**	**Q3**	**Maximum**	**Skewness**	**Kurtosis**	**MSSD**
Thermal conductivity	1.4108	0.4087	0.1670	28.9700	0.4830	1.1713	1.4015	1.7535	2.1510	− 0.4200	− 0.0200	0.1085
Thermal diffusivity	0.6714	0.1889	0.0357	28.1300	0.3150	0.5192	0.6905	0.8125	1.0000	− 0.3500	− 0.7200	0.0140
Specific heat	2.1030	0.5886	0.3465	27.9900	1.2660	1.7480	2.0105	2.2733	4.5470	2.0600	6.7800	0.2632
Temperature	27.7690	2.4400	5.9520	8.7900	23.1400	25.7750	28.6650	29.5470	32.3000	− 0.4000	− 0.9200	2.5290
Moisture content	0.1889	0.0233	0.0005	12.3200	0.1460	0.1683	0.1960	0.2090	0.2260	− 0.2900	− 1.1500	0.0001

Note: StDev = standard deviation; CoefVar = coefficient of variation, Q1 = first quartile, Q3 = third quartile; MSSD = mean of the squared successive differences.

## 2 Experimental design, materials and methods

The understanding of the thermal properties of soil is very important in agricultural science. This is because there is exchange of heat at the soil surface. The availability of the dataset on soil thermal properties would help in the improvements of wider applications of the heat of soil and modelling of the water transport in the soil. The availability of these dataset would also help in the understanding of seed germination and crop yield. Several works have been carried out on the various applications of thermal properties of soil [15], [16]], [17], [18], [19].

### 2.1 Field survey and laboratory analysis

**Fig. 1:**
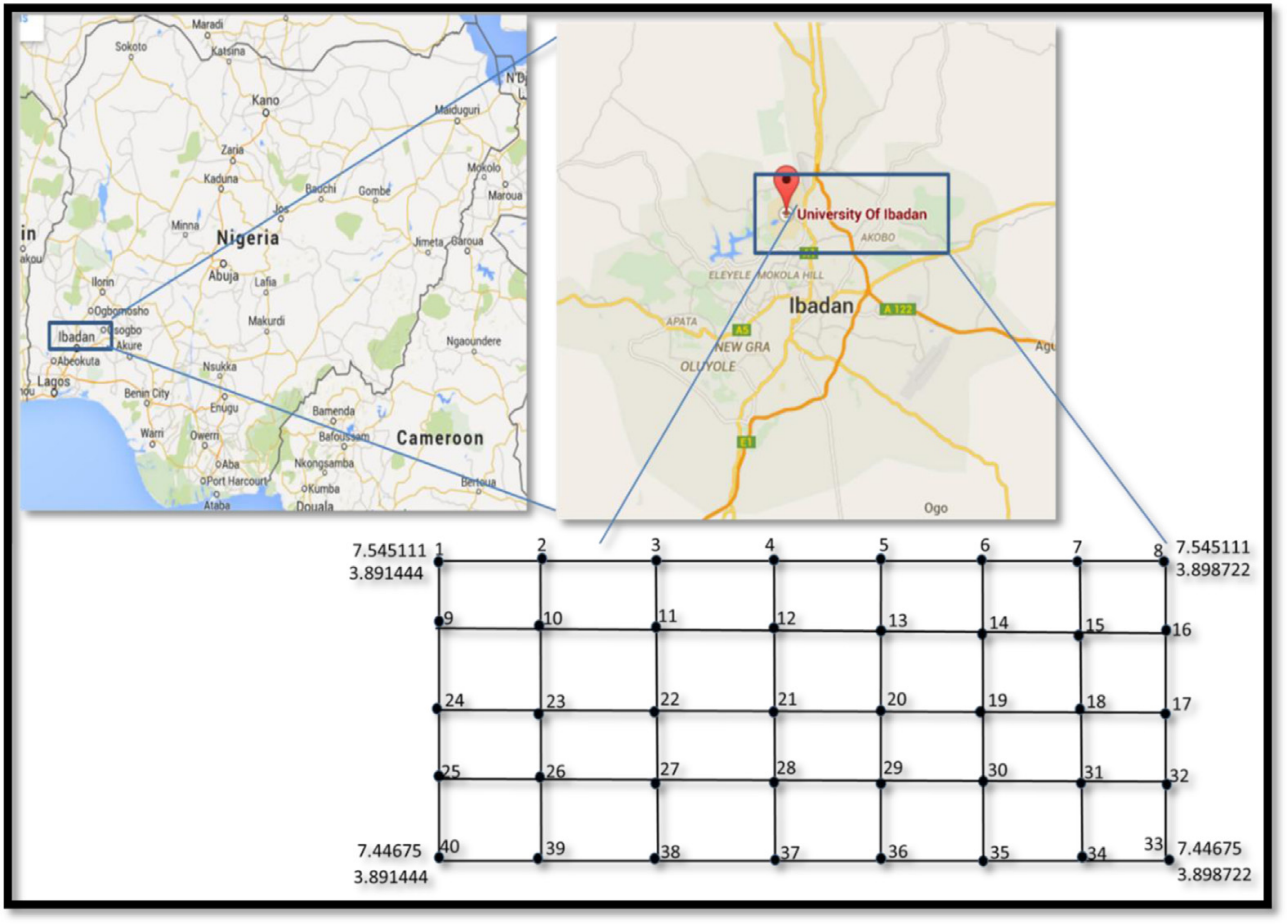
Map of the study area showing the sampling points.

**Fig. 2:**
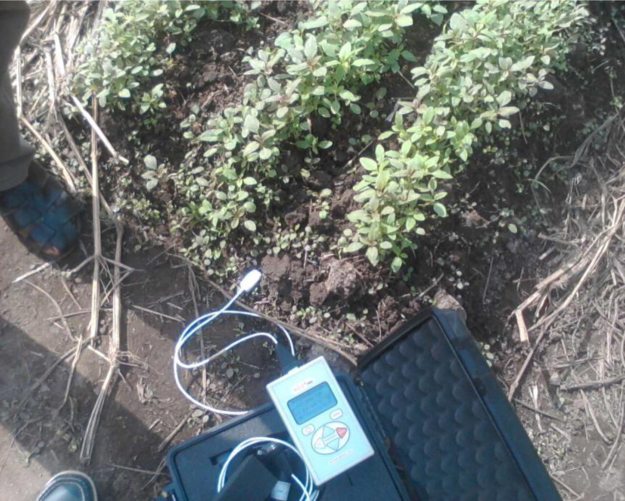
KD-2 Pro thermal analyzer.

The location of study is an agricultural farm land within the University of Ibadan campus. It is situated between latitude 7°26′.8020′′ – 7°26′.9320′′ and longitude 3°53′.7230′′ – 3°54′.0000′′, southwestern Nigeria (Fig. 1). The distribution of the sampling points is also presented in Fig. 1. The thermal properties were acquired using a thermal analyzer termed KD-2 *Pro* (Fig. 2). The KD2 Pro is a fully portable field and laboratory thermal properties analyzer. This probe makes use of transient line heat source technique in the determination of thermal properties of materials. A small dual-needles sensor called SH-1 was used for the measurements. The sensor measures the thermal conductivity, thermal diffusivity, volumetric specific heat and temperature of materials. This sensor uses the heat pulse methodology to generate dependable soil thermal conductivity and thermal diffusivity values. It also estimates the volumetric specific heat using a nonlinear least square procedure. SH-1 sensor is 30 mm long and 1.28 mm in diameter, and the spacing between the two needles is 6 mm.

Field sampling design was conducted prior to the data acquisition on the field and random sampling technique was adopted. The surface of the ground was scooped before measurements to remove the effects of top soil on the acquired data. The thermal sensor was calibrated using a white plastic cylinder (a two-hole Delrin block). This was done with a view to test the functionality of the sensor [17], [20] in order to ascertain that the sensor is operating according to the prescribed specifications. The Delrin block has two pre-drilled holes where the sensor was inserted. We then allowed it to calibrate for about 15 min before taking measurements. In order to measure thermal properties using the KD-2 *Pro*, SH-1 sensor was connected to the KD-2 *Pro* and was turned on. As part of the percussion for effective data acquisition, the sensor was placed into the soil correctly, maintaining the dual needle sensor to be parallel to each other while inserting the sensor into the soil. The probe was turned on and the thermal properties (conductivity, diffusivity and specific heat) measurements were conducted. Also, after taking the first measurement, the probe was then allowed to rest for more than 15 min before taking subsequent readings. This time is called measurement interval, which allows thermal gradients to dissipate (i.e. for equilibration between readings).

Forty sample points were considered for thermal properties measurements while soil samples were collected at these points to determine their moisture contents in the laboratory. These soil samples were put in polythene bags and stored in a cool dry place after which necessary laboratory analyses were carried out on them. Moisture contents were determined in the laboratory following the standard reference of AASHTO T265 [14].

### 2.2 Descriptive statistics

**Fig. 3:**
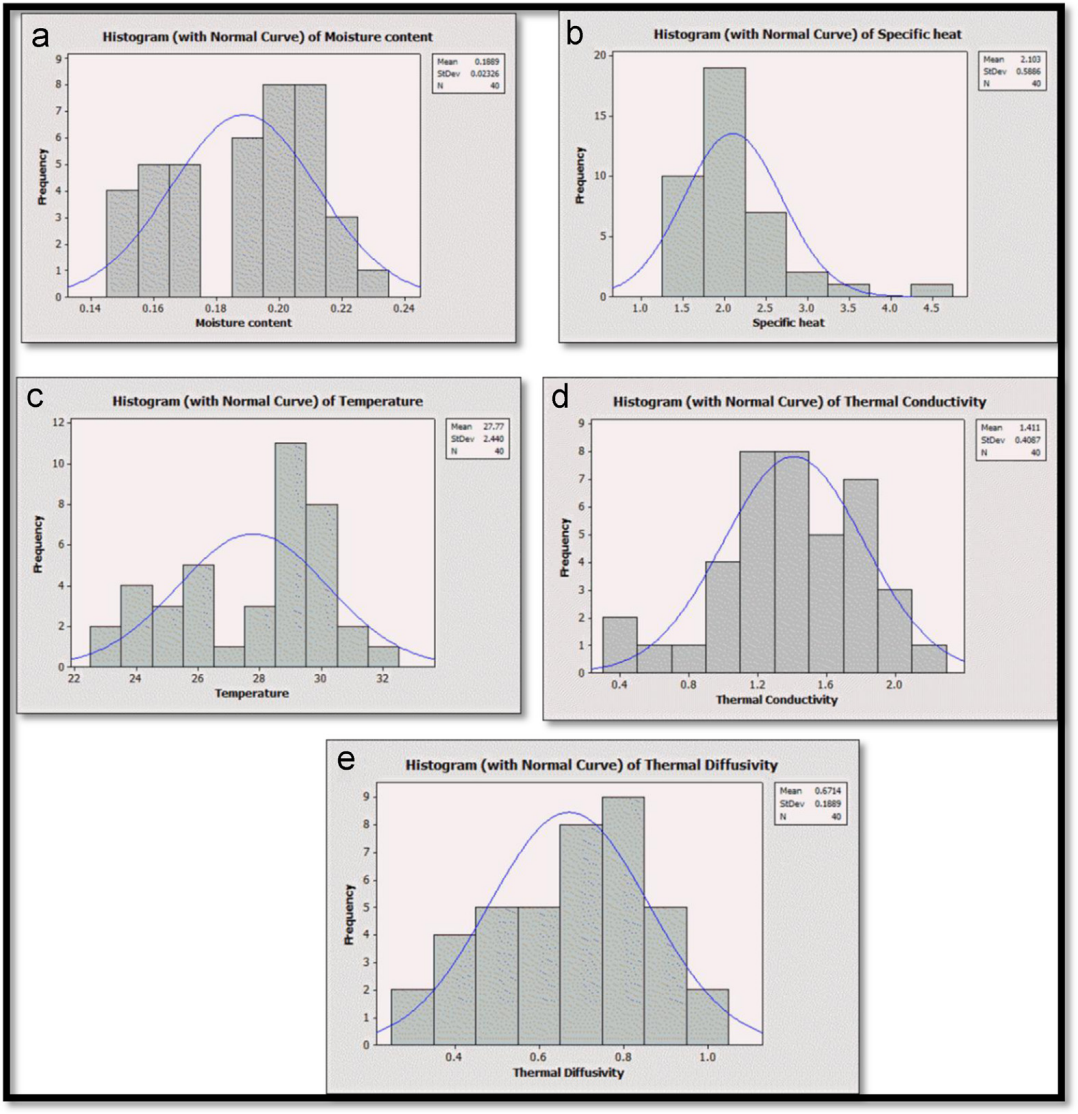
Histograms and normal curves (a) Moisture content (b) Specific heat (c) Temperature (d) Thermal conductivity (e) Thermal diffusivity.

The detailed descriptive statistics which provide basic statistical information about the measured thermal properties and moisture contents are presented in Table 2. The histogram plots indicate the statistical distribution of each measured properties as shown in Fig. 3.
